# Microbial Symbionts in Insects Influence Down-Regulation of Defense Genes in Maize

**DOI:** 10.1371/journal.pone.0011339

**Published:** 2010-06-28

**Authors:** Kelli L. Barr, Leonard B. Hearne, Sandra Briesacher, Thomas L. Clark, Georgia E. Davis

**Affiliations:** 1 Biological Sciences, Florida Gulf Coast University, Fort Myers, Florida, United States of America; 2 Plant Sciences, University of Missouri, Columbia, Missouri, United States of America; 3 Monsanto, St. Louis, Missouri, United States of America; University of Leeds, United Kingdom

## Abstract

*Diabrotica virgifera virgifera* larvae are root-feeding insects and significant pests to maize in North America and Europe. Little is known regarding how plants respond to insect attack of roots, thus complicating the selection for plant defense targets. *Diabrotica virgifera virgifera* is the most successful species in its genus and is the only *Diabrotica* beetle harboring an almost species-wide *Wolbachia* infection. *Diabrotica virgifera virgifera* are infected with *Wolbachia* and the typical gut flora found in soil-living, phytophagous insects. *Diabrotica virgifera virgifera* larvae cannot be reared aseptically and thus, it is not possible to observe the response of maize to effects of insect gut flora or other transient microbes. Because *Wolbachia* are heritable, it is possible to investigate whether *Wolbachia* infection affects the regulation of maize defenses. To answer if the success of *Diabrotica virgifera virgifera* is the result of microbial infection, *Diabrotica virgifera virgifera* were treated with antibiotics to eliminate *Wolbachia* and a microarray experiment was performed. Direct comparisons made between the response of maize root tissue to the feeding of antibiotic treated and untreated *Diabrotica virgifera virgifera* show down-regulation of plant defenses in the untreated insects compared to the antibiotic treated and control treatments. Results were confirmed via QRT-PCR. Biological and behavioral assays indicate that microbes have integrated into *Diabrotica virgifera virgifera* physiology without inducing negative effects and that antibiotic treatment did not affect the behavior or biology of the insect. The expression data and suggest that the pressure of microbes, which are most likely *Wolbachia*, mediate the down-regulation of many maize defenses via their insect hosts. This is the first report of a potential link between a microbial symbiont of an insect and a silencing effect in the insect host plant. This is also the first expression profile for a plant attacked by a root-feeding insect.

## Introduction

*Diabrotica virgifera virgifera* LeConte (Coleoptera: Chrysomelidae) or the western corn rootworm (WCR), are significant pests to maize in North America and Europe. Larval WCR feed below ground on maize root tissue while the adults feed on above ground tissue. WCR inhabit the largest geographical range of all rootworm species and, due to their close association with maize; their populations are more abundant and often supplant sympatric rootworm species. WCR are unique among rootworm species and other insect pests in that they have repeatedly surmounted control measures in far fewer generations than other crop pests [1]–[5].

Under normal circumstances, plants recognize and respond to insect attack through a variety of physiological, biochemical and molecular responses which include the release of volatiles and the production of proteins or metabolites that hinder the biology of the offending insect. These defenses can be classified into three categories which include bolstering of cell wall defenses, production of phytoalexins and the production of pathogenesis-related (PR) proteins. Cell wall defenses include strengthening of cell walls to prevent infection or deter feeding as well as senescence and lignification to trap microbes or make tissue less palatable to herbivores. Phytoalexins include almost every toxic chemical produced following insect or microbial attack. These chemicals include volatiles as well as salicylic acid and jasmonic acid related products. PR-proteins are unique from phytoalexins in that they are usually encoded by a single gene and are independent of pathways [6]. PR-proteins are non-detectable in healthy tissue and exhibit increased levels following microbial or insect attack [6].

Insect attack of maize normally results in the up-regulation of lipoxygenase (LOX), proteinase inhibitors, hydroperoxide lyase, PAL, methyl salicylate, methyl jasmonate, and a variety of volatile organic compounds [7]. These products can affect the insect by inducing the production of PR proteins that hinder the biology of the insect. Many of these products also defend the plant indirectly as well by catalyzing the production of compounds that attract natural enemies or signal neighboring plants of impending attack [8].

During recent years, microarrays have been used to identify genes specific for plant defense against insect attack [9], [10]. Results of these studies indicate that plants coordinate defense gene expression through various biochemical pathways and may be dependent on individual modes of attack. These studies, as with most plant response to insect studies, evaluated the response of leaf tissue and not root tissue.

It is accepted that insects are hosts to countless microbes with WCR being no exception. Little is known of the microbiota for the genus *Diabrotica*. Isolated populations with *Wolbachia* infections have been observed in *Diabrotica barberi* [11] and a small population of *Diabrotica undecimpunctata* has been shown to be infected with a unique strain of spiroplasma [12]. A profile of the gut flora of *Diabrotica balteata* has been described [13] and is reasonable to assume that WCR harbor similar infections given that both species have similar life cycles and biology. WCR beetles have been shown to harbor only *Wolbachia* [11]. Enterobacteria are limited to the digestive tract of the insect [14]; they are not heritable and must be acquired for each generation [15]. WCR acquire enterobacteria upon eclosion from the soil and plants upon which they feed and the bacteria are thought to aid in digestive processes [13]. It is possible to eliminate some enterobacteria via antibiotic treatment and subsequent culture in an aseptic environment [15]. Unfortunately, WCR cannot be reared aseptically as the larvae require a diet of corn root tissue and a soil-based environment [16] thus experiments with naive WCR are not currently possible. *Wolbachia* are intracellular bacteria and can be found throughout the body including the salivary glands, though concentrations are highest in reproductive tissues [17]. Unlike enterobacteria, *Wolbachia* are acquired through cytoplasmic inheritance. *Wolbachia* have been identified in several rootworm species including WCR but not in less successful species which are closely related to and sympatric with WCR [11].

To answer if *Wolbachia* affect the regulation of maize defenses, WCR were either treated with tetracycline or not for three generations and then both populations were reared for several generations under identical conditions. *Wolbachia* infection status was verified via PCR at each generation and prior to use in experiments. The presence of enterobacteria in the larval WCR was verified using universal primers which were kindly provided by Dr. Roger Stich (University of Missouri, Columbia). A microarray experiment was then performed in which direct comparisons were made between the response of maize to the feeding of antibiotic treated and untreated WCR. The data show that WCR that were not treated with antibiotics caused down-regulation of most plant defense genes while WCR that were treated with antibiotics induced up-regulation of the same defense genes.

## Results

A microarray experiment was performed using the Maize Oligonucleotide Array (www.maizearray.org). Three treatments with three biological replicates each of WCR with antibiotics, WCR without antibiotics and a non-insect control were evaluated. Larval WCR were allowed to feed on maize root for 24 hours, after which root tips were collected and assayed. A loop design with a dye-swap was employed which allowed for 18 total comparisons. An F-test for statistically significant difference between treatments within each probe and t-tests for between treatment comparisons within each probe were calculated. The calculated p-values from the t-tests were used to order the probes into a list for further exploration. Probes with a p-value greater than 0.5 were not considered statistically significant.

Of over 57 thousand oligos represented on the microarray, 23.8% (13,701) of the control treatment and 37.7% (21,660) of the untreated WCR treatment displayed statistically significant differential expression. The antibiotic treated WCR exhibited a significant change in gene expression for 39.1% (22,490) of the genes contained on the microarray. 29,562 (51.2%) displayed statistically significant differential expression for all three treatments. Of these 29,562 genes, 68% (20,119) and 63% (18,792) displayed significant differential expression when the antibiotic treated WCR treatment was compared to the control and untreated WCR treatments respectively. The untreated WCR treatment showed that 41% (12,393) of the 29 thousand genes had significant changes in gene expression when compared with the control treatment.

**Figure 1:**
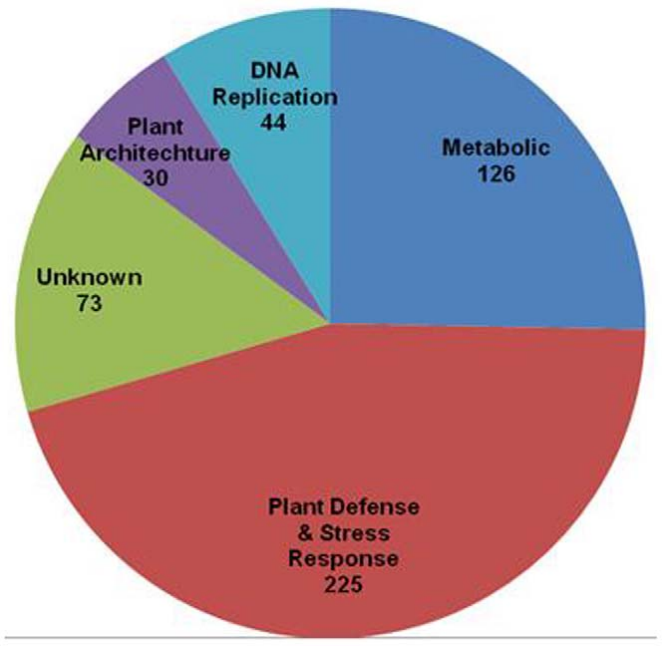
Classification of 500 genes with the most significant differential expression. 500 genes with the most significant differential expression were categorized using a combination of gene annotation, gene ontology and scientific publication. 225 genes were related to plant defense and stress response, 126 genes were associated with metabolic processes, 30 were categorized as being involved with plant architecture and/or development, 44 genes were associated with DNA replication and 73 genes could not be classified as their functions are as of yet, unknown.

**Figure 2:**
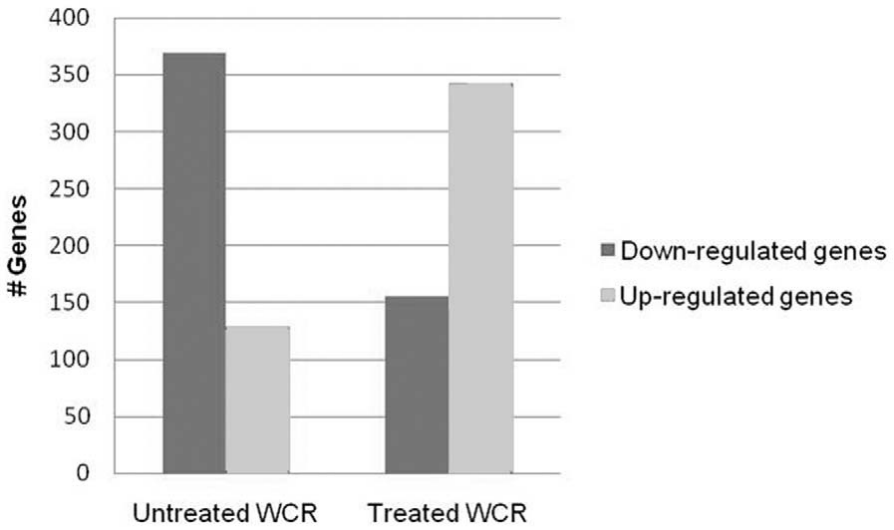
Expression pattern of untreated and antibiotic treated WCR. The relative expression of the untreated and antibiotic treated WCR treatments was evaluated in respect to the control treatment. The untreated WCR treatment exhibited a down-regulation for 369 of the 500 genes. The antibiotic treated WCR showed and up-regulation of 343 of the 500 genes.

**Figure 3:**
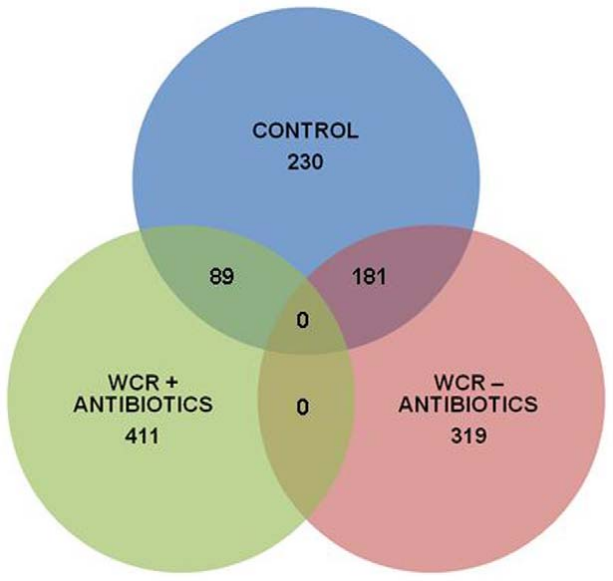
The relationship of the 500 most significant genes between the 3 treatments. A Ven diagram representation of the distribution of the 500 most significant genes indicates that no genes displayed similar expression for all 3 treatments. The control treatment shared similar expression of 89 genes with the treated WCR treatment and 181 genes with the untreated WCR treatment. The WCR treatments did not share similar expression for any of the 500 genes.

Generally speaking, the antibiotic treated WCR treatment tended to be down regulated in expression in relation to the control and untreated WCR treatments. The untreated WCR treatment tended to exhibit an increase in genetic expression in relation to the control and antibiotic treated WCR treatments. For this paper, analysis was limited to the 500 genes displaying the most statistically significant change in differential expression when all three treatments were compared to each other (Table S1). These 500 genes were then classified using a combination of gene annotation, gene ontology and published research. Of these 500 genes, 45% (225 genes) are associated with plant defense and stress response, 25% (126 genes) are related to metabolic processes, 15% (73 genes) are of unknown function, 6% (30 genes) are involved with plant architecture and 9% (44 genes) are associated with DNA replication (Figure 1). Seventy-four percent (369 genes) of the differentially expressed genes for the untreated WCR treatment were down-regulated in relation to the control and antibiotic treated WCR treatments (Figure 2). Sixty-nine percent (343 genes) of the 500 genes were up-regulated for the antibiotic treated WCR in relation to the control and untreated WCR treatments (Figure 2). When the 3 treatments were compared to each other, all 500 genes analyzed were statistically significant in respect to each individual treatment (Figure 3). When the untreated WCR and control treatments were compared, the relative expression 181 of the 500 genes was statistically similar (Figure 3). When the antibiotic treated WCR and control treatments were compared to each other, the relative expression of only 89 of the 500 genes were statistically similar (Figure 3). When the antibiotic treated WCR and untreated WCR treatments were compared, none of the 500 genes exhibited statistically similar differential expression (Figure 3).

**Figure 4:**
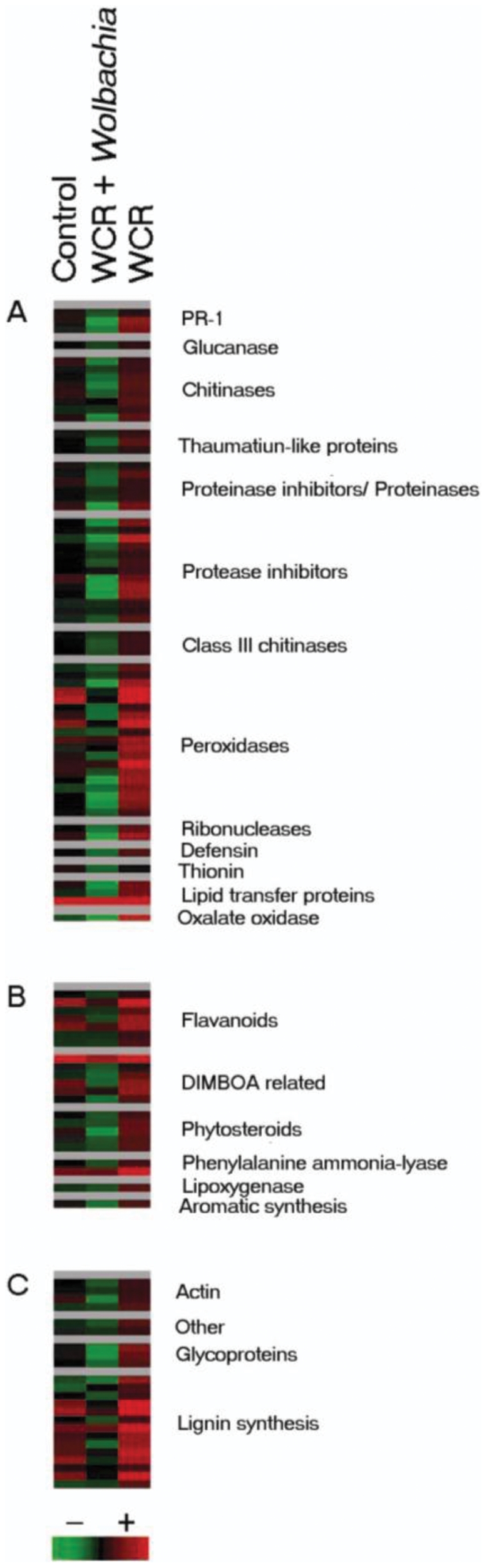
Expression profile of maize defense genes from TIGR Multiple Array Viewer. The expression profile of the data represented as a heat map illustrates that feeding by untreated WCR resulted in down-regulation of plant defense to levels below that of the non-feeding control and up-regulated following attack of antibiotic treated WCR. The column designated as WCR + *Wolbachia* represents WCR without antibiotic treatment. Likewise, the column designated as WCR represents WCR that were treated with tetracycline. (A) PR proteins, (B) Phytoalexins, (C) Cell wall associated factors. Green indicates gene down-regulation while red indicates gene up-regulation.

Analysis of microarray expression data shows a genome-wide suppression of maize defense genes following attack by untreated WCR (Table S2). The expression profile of the data represented as a heat map illustrates that feeding by untreated WCR resulted in down-regulation of all categories of plant defense to levels below that of the non-feeding control (Figure 4). The heat map also demonstrates that maize defense genes are up-regulated following attack of antibiotic treated WCR (Figure 4). Other genes coding for metabolic factors, plant architecture, and DNA replication were differentially expressed between all three treatments (Table S1).

Probes differentially expressed in the microarray study included genes coding for cell wall structure and defense, phytoalexins and 16 of 17 classes of PR proteins (Figure 4, Table 1, Table S2). Effected cell wall factors included lignins, actin and glycoproteins (Figure 4, Table 1, Table S2). Effected phytoalexins included, phytosteroids, flavonoids and hydroxamic acids (Figure 4, Table 1, Table S2). Differentially expressed PR proteins included PR-1 proteins, glucanase, chitinases, thaumatin-like proteins, ribonucleases, peroxidases, protease and proteinase inhibitors, defensins, thionins, lipid transfer proteins, oxalate oxidases and glycoproteins (Figure 4, Table 1, Table S2). Notably, several genes within each category were suppressed when attacked by untreated WCR.

**Table 1:** Differentially expressed maize defense probes.

Array I.D.	Control	WCR - tetracycline	WCR + tetracycline	Annotation
MZ00004486	0.28629	−0.70834	0.42204	Pathogenesis related protein-1
MZ00042168	0.27636	−2.00827	1.73191	Pathogenesis related protein-1
MZ00043035	0.29500	−1.26578	0.97078	Chitinase
MZ00000977	−0.14194	−0.37386	0.51580	Putative antifungal thaumatin-like protein
MZ00013547	−0.03908	−1.00381	1.04289	Thaumatin-like protein
MZ00044339	0.26643	−0.53451	0.26808	Cysteine proteinase inhibitor
MZ00035455	−0.13164	−0.87217	1.00381	Cysteine proteinase CP1
MZ00038348	0.43674	−0.92740	0.49066	Cathepsin B-like cysteine proteinase
MZ00002045	−0.09321	−0.34906	0.44227	Putative aspartic proteinase nepenthesin I
MZ00025326	−0.07839	−0.46348	0.54187	Aspartic proteinase
MZ00018372	−0.09187	−0.50369	0.59556	Class III chitinase RCB4
MZ00037339	0.03418	−0.64691	0.61273	Chitinase
MZ00021144	−0.10106	−0.48489	0.58595	Putative Peroxidase 1 precursor
MZ00022862	−0.20443	0.01207	2.05420	Class III peroxidase
MZ00033093	−0.01601	−1.55862	1.57463	Putative peroxidase P7X
MZ00027915	−0.05187	−1.12610	1.17797	Pathogenesis-related protein 10
MZ00028247	0.49832	−2.62245	2.12414	Putative aleurone ribonuclease
MZ00043949	0.08557	−1.05327	0.96771	Defensin 1 precursor
MZ00016209	0.00094	0.64830	0.00000	Thionin
MZ00003835	0.33676	−1.67161	1.33486	Putative lipid transfer protein
MZ00039775	0.10281	−0.48354	0.38073	Xylanase inhibitor protein I
MZ00036538	−0.41536	−2.10232	2.51768	Subtilisin/chymotrypsin inhibitor
MZ00025431	−0.03244	−0.73077	0.76321	Bowman-Birk type trypsin inhibitor
MZ00011113	0.01716	0.15841	0.69393	OTU-like cysteine protease-like
MZ00005428	−0.81427	0.01629	0.79798	4-hydroxycinnamic acid-CoA ligase
MZ00044023	−0.03263	−0.69551	0.72814	Cinnamoyl-CoA reductase
MZ00014292	1.32518	1.17489	3.59778	Phenylalanine ammonia-lyase
MZ00025513	−0.55650	−0.46633	1.02284	Cinnamic acid 4-hydroxylase
MZ00028764	0.07526	−0.46305	0.38778	Dihydroflavonol4-reductase
MZ00026160	0.79660	−0.60977	1.92247	Glutathione S-transferase GST 30
MZ00041712	−0.38460	−0.43680	0.82140	Glutathione S-transferase GST 8
MZ00015236	3.08190	2.10015	5.06733	UDP-glucosyltransferase BX9
MZ00012679	−0.27730	−0.95516	1.04017	Probable hydroquinone glucosyltransferase
MZ00000005	−0.39050	−0.67223	1.06273	Lipoxygenase
MZ00043996	−0.16248	−0.94616	1.10864	Bax inhibitor-1
MZ00032776	0.02366	−0.30412	0.28045	Putative disease resistance response protein

An example of some of the maize probes that were down-regulated when attacked by untreated WCR and up-regulated when attacked by antibiotic-treated WCR. The values in the columns represent the average relative expression for all replicates.

As a rule, defense genes of all three classes were down-regulated in maize when attacked by untreated WCR. However, several maize defense genes were up-regulated following attack of untreated WCR and could be classified as being involved with DNA replication and repair, gene silencing and microbial defense (Table S1, S2). There were several metabolic, signaling and architectural factors up-regulated after untreated WCR attack as well (Table S1). The expression status of 6 of the genes in question was verified via quantitative RT-PCR; the absolute quantifications support the microarray analysis (Table 2). The expression pattern for over 50 genes was verified via semi-quantitative PCR (Table S3); the data support the microarray results. WCR were checked for enterobacteria and *Wolbachia* via PCR. Evidence of *Wolbachia* was not found in antibiotic treated WCR; however enterobacteria were present in both antibiotic treated and untreated WCR.

**Table 2:** QRT-PCR of 6 differentially expressed genes.

		Treatment		
ID	Annotation	Control	WCR - tetracycline	WCR + tetracycline
MZ00044023	Cinnamoyl COH reductase	52.9	48.64	54.64
MZ00035455	Cystene protease 1	113.65	100.27	151.25
MZ00004041	PAL	30.14	20.38	80.34
MZ00039775	Xylanase Inhibitor	1.98	1.66	3.37
MZ00001590	AGO1	125.88	304.86	392.15
MZ00016076	Hec1	1.51	11.12	9.55

Absolute quantification values for 6 differentially expressed genes displayed as ng RNA. The expression pattern for the 6 genes reflects the results of the microarray.

**Figure 5:**
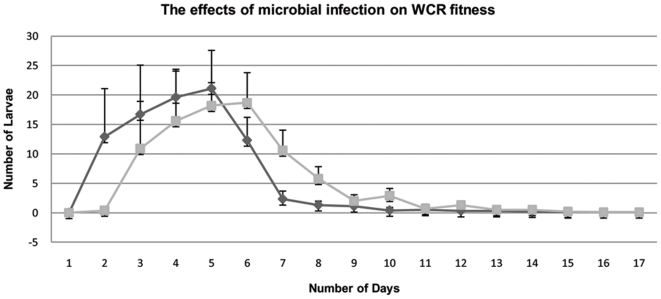
The effects of microbes on WCR fitness. A hatch assay was conducted in from an isolated sample of eggs; both colonies display a bell shaped hatch distribution in relation to time. T-tests for number hatched and rate of hatch are statistically not significant. Diamonds represent WCR that were treated with tetracycline while squares represent WCR without antibiotic treatment.

In order to identify the effect of *Wolbachia* on WCR survivability, a hatch assay was conducted in which the eclosed larvae from an isolated sample of eggs were counted over time. The antibiotic treated colony exhibited an 89% total hatch while the untreated colony displayed an 88% total hatch. A t-test comparing the percent hatch for both populations rendered a p-value of 0.61. Both colonies display a bell shaped hatch distribution in relation to time illustrating the similarities between the two colonies (Figure 5). A t-test of the hatch rate over time gave a p-value of 0.78 which further indicates the similarity between the two colonies (Figure 5).

**Figure 6:**
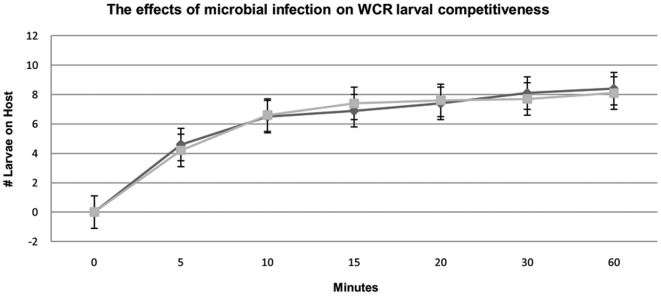
The effects of microbes on WCR larval competitiveness. A host location assay was conducted to determine the effect of microbial infection on WCR larval competitiveness. T-tests for number of WCR that were able to locate the host plant and rate of host location are statistically not significant. Diamonds represent WCR that were treated with tetracycline while squares represent WCR without antibiotic treatment.

In order to determine if *Wolbachia* infection affects WCR larval competitiveness, a host location assay was conducted. Because WCR larvae must locate a host plant within 24 hours of eclosion before mortality has significant effects [18], larvae that were 12 hours old or younger were used for the experiment. There was no significant difference in the ability of the larval WCR to find a maize seed between the treated and untreated populations. Eighty-four percent of the untreated WCR vs. 81% of the antibiotic treated WCR located a maize seed in 1 hour or less. A t-test comparing the total number of larvae able to locate the maize seed yielded 0.5413571 with a p-value of 0.54 indicating that microbial infection does not affect WCR larval competitiveness (Figure 6). A second t-test comparing the rate of host location between the two colonies yielded 0.750714 with a p-value of 0.75 further supporting the similarity between antibiotic treated and untreated WCR (Figure 6).

## Discussion

The inability of a plant to express certain genes can make it susceptible to attack from other factors which are not normally a threat. The transcription profile of the response of maize roots to WCR attack exhibited a specific pattern. Untreated WCR induced a down-regulation of maize defense genes in relation to the antibiotic treated WCR and the control treatments. Likewise, the antibiotic treated WCR induced an up regulation of maize defenses in relation to the untreated WCR and the control treatments.

The reinforcement of cell walls is the first line of plant defense which is repressed by untreated WCR feeding and increased by antibiotic treated WCR feeding. Lignin is a structural component of cell walls; during defense, lignin has been shown to accumulate around areas of attack and create a physical barrier against infection [19]. Cinnamoyl-CoA reductase and cinnamyl alcohol dehydrogenase catalyze the first steps in lignin synthesis; genes for both products were down-regulated in the presence of untreated WCR (Table 1). Since neonate WCR feed by burrowing inside the root, down-regulation of lignin associated products may indicate that the maize root is remaining palatable and/or digestible to the insect. Decreased amounts of lignin may make it easier for the larval WCR to burrow inside the root tissue.

Genes encoding glycoproteins were down-regulated when untreated WCR fed on maize. Decreased production of glycoproteins can make maize vulnerable to pathogens by weakening cell wall defenses [20]. Several actin-specific probes were down-regulated in the presence of untreated WCR. Studies indicate that the inhibition of actin results in cell wall permeablization through which both pathogenic and non-pathogenic microbes may pass [21]. Decreased expression of structural components could signify that either the maize tissue is being rendered more digestible for the insect or the cell walls are being weakened to facilitate microbial infection.

Several phytoalexin-related genes were down-regulated in maize following feeding by untreated WCR but up-regulated after antibiotic treated WCR (Table 1, Table S2). The data show that PAL was down-regulated after exposure to untreated WCR (Table 1); PAL marks the first committed step in flavonoid synthesis from which several defense products are derived. Flavonoid related products such as dihydroflavonol 4-reductase have been shown to confer increased resistance to bacterial pathogens [22]. Several Glutathione-S-transferase encoding genes on the microarray were down-regulated after untreated WCR feeding (Table 1, Table S2) which may suggest a problem in the plant with detoxification of toxic substances from the insect or bacteria. Probes which code for glucosyltransferases and other products on the hydroxamic acid pathway were down-regulated after exposure to untreated WCR. Maize produces the hydroxamic acid DIMBOA which has been shown to have deleterious effects on WCR larvae [23].

Untreated WCR induced the down-regulation of a shikimate kinase gene (Table S2) that is involved in the synthesis of aromatic compounds which can both deter insects from feeding and attract natural enemies [24]. Untreated WCR feeding resulted in the down-regulation of probes encoding lipoxygenase (LOX) and LOX related metabolites (Table S2). Down-regulation of LOX causes decreased production of oxylipins and protease inhibitors as well as increased insect attack and colonization by insects which are not normally associated with the plant [25]. LOX metabolites such as jasmonic acid and hexanal have been shown to facilitate volatile production in maize [26]. Other probes coding for volatiles such as indole, ethylene, beta-caryophyllene and sequiterpenes were not significantly different from the non-feeding control in maize attacked by untreated WCR.

Sixteen of seventeen classes of PR proteins were down-regulated in maize when attacked by untreated WCR while being up-regulated when attacked by antibiotic treated WCR. It is noteworthy that the literature shows that most PR proteins are evidenced to be specific for microbial and not insect attack [27]–[30]. Thaumatin-like proteins have been linked with fungi as have most chitinases. Plants with decreased expression of thaumatin-like proteins have been shown to be more susceptible to fungal attack [31]; while increased levels of some thaumatin-like proteins confer resistance against several classes of fungi [32]. Increased yields of cysteine proteinases in maize are responsible for gut proteolysis in WCR [33]. Chitinases have been shown to inhibit *A. flavus* and *Fusarium* colonization in maize [34]. Defensins have also been shown to have antimicrobial properties in maize [35]. Perhaps these products act on the insect midgut in the same manner as they perform on fungal membranes as the insect midgut is composed of the same materials as fungal cell walls. Thaumatin-like proteins and chitinases may be an unrecognized class of defense products which have significant contributions to plant defense against insect feeding.

Interestingly, two antimicrobial defense genes, a translational elongation factor EF-TuM which is involved in microbial resistance [36] and an N-carbamyl-L-amino acid amidohydrolase which it the first step of an antibiotic synthesis pathway [37] were significantly up-regulated in maize in response to attack by untreated WCR (Table S2).

The results beg the questions, “What microbes are causing changes to maize gene expression?” Though there are countless species of microbes that may infect WCR, given the experimental system and biological requirements of WCR, it is not possible to investigate each of the thousands of species of potential WCR-infecting microbes. Attempts were made to introduce *Wolbachia* into the cured WCR population using both injection and oocyte permeablezation techniques but were unsuccessful in producing fertile adults. However, the experimental design and features of the data allow for the elimination of most microbes. A microbe that could contribute to the expression profile would have to possess specific characteristics. First, it would have to be heritable as both WCR populations were reared under identical conditions yet, after several generations only the tetracycline-treated population was able to elicit a defense response in maize. Second, the microbe would have to be susceptible to tetracycline as the data indicate that once WCR are treated with tetracycline, maize is able to up-regulate a defense response. Third, the microbe would have to maintain a species-wide infection as the expression data for the untreated WCR population is statistically similar to the response of maize to WCR which originated from various locations across the Midwest (unpublished data). Fourth, the microbe would have to be non-pathogenic to WCR as the fitness assays show that WCR are not suffering any ill effects from infection (Figure 5, Figure 6). The only microbe known to this experimental system that can adhere to these standards is *Wolbachia*.

*Wolbachia* are gram negative, obligate intracellular alpha-proteobacteria which share a monophyletic relationship with Rickettsia and Ehrlichia [38]. Several studies have shown that Ehrlichia and Rickettsial bacteria silence mammalian immune response in order to establish infection and facilitate symbiosis [39]. It has been shown that *Wolbachia* from filarial worms can mediate genetic responses in humans and other mammals [40]–[45]. *Wolbachia* can be found in up to 75% of all insect species [46]. Aside from infecting many agronomic pests, *Wolbachia* are associated with most arbovirus vectors and all forms of filarial disease [47]. *Wolbachia*, though parasitical in nature, are described as symbionts since their mode of cyptoplasmic transmission has caused them to develop strategies that increase the fitness of the female host [48]. WCR are naturally infected with a distinct strain of *Wolbachia pipientis* that induces cytoplasmic incompatibility which is thought to serve as a reproductive barrier between sympatric species of rootworm beetles [49].

The microarray data show that microbes can override the effects of insect elicitors on the plant which may allow WCR to utilize the host plant more effectively than other insects. The symbiosis of microbes and their various hosts display a variety of interactions that can be beneficial or harmful to one or both organisms. Assays on antibiotic treated and untreated WCR larval competitiveness and fertility indicate that *Wolbachia* and, perhaps, other unidentified microbes have integrated into WCR physiology without inducing negative effects. This indicates that WCR and the said microbes share, at the very least, a commensal association.

Reports have been made of insects eliciting plant responses similar to pathogens however; these studies did not implicate *Wolbachia* or other microbes as the causal agent [10], [50], [51] even though the insects in these studies harbor *Wolbachia*. Data from these studies is similar to our own in that typical insect defenses and oxidative bursts are missing. Clearly, a reassessment of paradigms involving plant-insect interactions is necessary and further investigation of microbial-associated tritrophic interactions is warranted.

## Materials and Methods

### Insect Culture

All *Wolbachia* infected WCR used in these experiments were kindly provided by Dr. Bruce Hibbard at the USDA-ARS in Columbia, MO and from the USDA-NGIRLS in Brookings SD. A *Wolbachia*-free colony was generated from WCR obtained from these infected population and field-caught adults. WCR were cured as described elsewhere [49] except that selection occurred for three generations instead of two. The newly hatched WCR of the fourth generation were sexed and segregated upon emergence from the soil. One leg from each adult was removed and assayed for the presence of *Wolbachia*. Adults that were negative for *Wolbachia* were placed in a community cage and allowed to mate randomly with other non-infected individuals. The cured WCR were then reared as according to standard methods using the same soil and diet as the infected colony [16]. The cured colony was allowed to reproduce for over a year producing 5–6 generations. In an effort to reduce the effects of a bottlenecked population, over 1000 individuals over a 6 month period were incorporated into the primary selection. Infection status of the WCR was verified via PCR with *Wolbachia* specific primers coding for a 16s ribosomal RNA fragment [52]. The presence of enterobacteria was verified via PCR with universal primers which were kindly provided by Dr. Roger Stich in the department of Veterinary Pathobiology at the University of Missouri. Infection status was verified prior to each experiment. Once hatching had commenced, only vigorously moving larvae were selected. Larvae were selected with a small camel hair paintbrush and placed into a standard Petri dish. Viability of the selected larvae was verified by visualization with a dissection microscope. Any injured larvae were replaced with healthy larvae. Larvae were placed at the base of the maize plants by rinsing the Petri dish with water.

### Plant Tissue Preparation

For the microarray experiment, a CRW3 (S1)C6 (Reg. No. GP-553, PI 644060) which had been selectively bred for WCR resistance was chosen [53]. This line is segregating and was chosen for study as more alleles would be present as opposed to a standard hybrid. Our goal was to observe the effects of *Wolbachia* on as many alleles as possible. Maize plants were grown in a growth chamber with conditions set at a 14 hour photoperiod and 10 hour scotoperiod. Both incandescent and florescent lights were used and a light level of 650–700 microeinsteins was maintained. Temperatures were set at 28°C for the photoperiod and 22°C for the scotoperiod with humidity at 60% and 80% respectively. In order to mimic field conditions, maize seed was planted in soil containing 1% nitrogen, 0.5% potassium and 0.5% phosphorus fertilizer. Plants were grown in 360 ml plastic drinking cups which were perforated for drainage at the bottom of the cup. Plants were grown to the V3 stage where they were subjected to their respective treatments.

### Tissue Collection for Microarrays and RT-PCR

Three treatments with three biological replicates per treatment were assayed. The treatments included: maize with untreated WCR, maize with untreated WCR, maize with no WCR. For each replicate, 75 plants per treatment were pooled into a single sample. For the WCR feeding treatments, 50 neonate larvae were placed at the base of the plant. Root tissue was collected 24 hours post-infestation. Control plants were not infested with either type of WCR. Tissue from all treatments was collected in the dark with the use of a green light. In 30 seconds or less, soil was dislodged from the roots, the roots were rinsed in room temperature water and then a centimeter of tissue from a seminal root tip was excised with a scalpel and placed into liquid nitrogen.

### Microarray Experimental Design, Hybridization and Data Anaylsis

For this experiment, the Maize Oligonucleotide Array was used (www.maizearray.org). The Maize Oligonucleotide Array contains 57,452, 70-mer oligonucleotides that encompass the maize genome. A standard loop design for the groups; non-feeding control, untreated WCR feeding and antibiotic treated WCR feeding was employed. Each of the pair-wise comparisons was replicated three times using the three biological replicates of maize root tissue. A dye swap was also performed. Thus, there were a total of 36 experimental samples hybridized on 18 microarrays. RNA extraction, amplification and hybridization were performed according to the protocols of the Maize Oligonucleotide Array Project (www.maizearray.org/maize_protocols.shtml). An extra RNA cleanup was performed after the initial RNA extraction in order to remove any residual sugars from the sample. Following hybridization, the slides were washed, dried and immediately scanned. A GenePix 4000B Axon scanner (Molecular Devices Corporation, Sunnyvale, California) was used. Slides were prescanned and a probe intensity curve with a count ratio of 1.0 +/− 0.1 was obtained before a final image was acquired. GenePix Pro version 6.0 software was employed for slide normalization and spot-calling.

The slides were first scanned using a pre-scan self calibration procedure that set the photo-multiplier gain for optimal dye resolution. The optical intensities were then log transformed and the data analyzed using a two stage mixed linear model. In the first stage the across array fixed effects; panel, dye, treatment and mixed model effects; array and dye within array were modeled. The residuals were then modeled by probe using a fixed effect for dye and treatment. An F-test for statistically significant difference between treatments within each probe and t-tests for between treatment comparisons within each probe were calculated. The Wilcoxon rank sum test was also calculated for each probe to add confidence for significant results. The calculated p-values from the t-tests were used to order the probes into a list for further exploration. Data for this microarray experiment are MIAME compliant and have been deposited with ArrayExpress accession numbers are E-MEXP-2391 and E-MEXP-2392. Accession number E-MEXP-2391 corresponds to slide A and accession number E-MEXP-2392 corresponds to slide B.

### Quantitative and semi quantitative RT-PCR

To confirm the results of the microarray analysis, the relative expression of 6 selected probes was determined by quantitative real-time PCR. Differentially expressed probes were selected from the genes assayed on the oligoarray. Sequence specific primers were designed using PrimerQuest from IDT SciTools (http://www.idtdna.com/Scitools/Applications/PrimerQuest/Default.aspx/).

iScript One-Step qRT-PCR Kit with SYBR Green (BioRad, Hercules, CA) was used. Half reactions were performed with 30 ng of total RNA per sample. A standard curve was set on each plate. RNA from the control treatment and housekeeping primers were used. Standard curve concentrations were set at 100 ng, 50 ng, 25 ng, 12 ng, 6 ng and 3 ng of total RNA. A no template control was set on each plate. An ABI7000 real-time PCR system (Applied Biosystems) was employed for mRNA quantification and verification of the microarray analyses. Cycles were programmed according to manufacturer's specifications in the iScript One-Step qRT-PCR Kit with SYBR Green kit. A melting curve analysis was added at the end of each analysis. The expression of several of the 50 selected genes was also verified by semi quantitative RT-PCR via PCR amplification of cDNA and gel electrophoresis. A list of primers and results of the semi-quantitative RT-PCR have been included with the supplementary information (Table S3).

### Hatch Assay

*Wolbachia-*positive WCR or *Wolbachia-*negative WCR were allowed to oviposit in sterile oviposition plates for three days. Eggs were washed from the oviposition medium using a fine sieve under running water and examined under a microscope. One hundred viable eggs were placed in a standard Petri dish lined with Whatman filter paper that was kept damp with sterile water. The Petri dishes were sealed with parafilm and allowed to incubate at 25°C. Hatch counts were taken every 24 hours. Data was collected until seven days had passed without hatch. Eclosed larvae were counted and removed from the sample. Ten biological replications of 100 eggs each were performed for both *Wolbachia-*positive WCR and *Wolbachia-*negative WCR. Data were plotted to visualize a hatch curve. Statistical analysis was performed using a Student's t-test.

### Host Location Assay

Mo17 maize seed that had been imbibed for 24 hours was allowed to germinate for four days at 25°C. The seed was placed at the center of a standard size Petri dish that has been lined with moistened Whatman filter paper. Since moisture levels can influence larval movement, equal amounts of sterile water were added to each dish to maintain adequate moisture levels. Both *Wolbachia-*positive WCR and *Wolbachia-*negative populations were tested at the same time. Because larvae usually emerge from the oviposition medium prior to collection, samples can be biased towards individuals with increased fitness. Therefore, neonate WCR larvae were collected from eggs that had been washed free of oviposition medium and incubated as in the hatch assay. Ten neonate larvae were collected with a camel hair brush and placed within the outer first centimeter of a dish containing a germinated maize seed. Any injured larvae were removed and replaced and the dish was then sealed with parafilm. Larvae were timed to quantify how long it took each individual to locate the host plant. Counts were taken every five minutes for an hour. Host location was observed when larvae ceased searching and located the maize root. Ten replications were performed for each insect type. The data were analyzed using a Student's t-test.

## Supporting Information

Table S1 The 500 genes with the most significant differential expression.
(0.09 MB XLS)

Table S2 Maize defense genes with significant differential expression.
(0.05 MB XLS)

Table S3 Primers used for QRT-PCR and semi quantitative RT-PCR.
(0.05 MB XLS)
